# Supplementary material for: Multi‐dataset identification of innovative feature genes and molecular mechanisms in keratoconus
Source: J Cell Mol Med. 2024 Sep 19;28(18):e70079. doi: 10.1111/jcmm.70079 (PMC11412914; doi:10.1111/jcmm.70079)
Supplement: Supplementary file 2 — Material S2. [file JCMM-28-e70079-s002.zip › 7.GSEA analysis/2.ATOH7/KEGG_TIGHT_JUNCTION.html]

Details for gene set KEGG\_TIGHT\_JUNCTION[GSEA]

|  || Dataset | ATOH7.group.cls#h\_versus\_l |
| Phenotype | group.cls#h\_versus\_l |
| Upregulated in class | h |
| GeneSet | KEGG\_TIGHT\_JUNCTION |
| Enrichment Score (ES) | 0.36877984 |
| Normalized Enrichment Score (NES) | 1.3406858 |
| Nominal p-value | 0.10207939 |
| FDR q-value | 0.35152668 |
| FWER p-Value | 0.941 |
Table: GSEA Results Summary

  

Fig 1: Enrichment plot: KEGG\_TIGHT\_JUNCTION      
 Profile of the Running ES Score & Positions of GeneSet Members on the Rank Ordered List

  

| SYMBOL | TITLE | RANK IN GENE LIST | RANK METRIC SCORE | RUNNING ES | CORE ENRICHMENT || 1 | AKT3 | na | 9 | 1.043 | 0.0547 | Yes |
| 2 | JAM2 | na | 98 | 0.775 | 0.0930 | Yes |
| 3 | MRAS | na | 141 | 0.733 | 0.1304 | Yes |
| 4 | MYL7 | na | 272 | 0.636 | 0.1602 | Yes |
| 5 | MAGI2 | na | 788 | 0.481 | 0.1706 | Yes |
| 6 | JAM3 | na | 846 | 0.472 | 0.1938 | Yes |
| 7 | PRKCE | na | 1002 | 0.447 | 0.2129 | Yes |
| 8 | MYL10 | na | 1159 | 0.425 | 0.2308 | Yes |
| 9 | CLDN2 | na | 1390 | 0.398 | 0.2451 | Yes |
| 10 | EPB41L2 | na | 1439 | 0.392 | 0.2644 | Yes |
| 11 | IGSF5 | na | 1544 | 0.382 | 0.2815 | Yes |
| 12 | MYH8 | na | 1589 | 0.378 | 0.3001 | Yes |
| 13 | HCLS1 | na | 2137 | 0.334 | 0.3019 | Yes |
| 14 | CLDN14 | na | 2270 | 0.325 | 0.3152 | Yes |
| 15 | PRKCB | na | 2281 | 0.324 | 0.3320 | Yes |
| 16 | PARD6B | na | 2333 | 0.321 | 0.3474 | Yes |
| 17 | ACTN2 | na | 2998 | 0.281 | 0.3430 | Yes |
| 18 | MAGI1 | na | 3170 | 0.272 | 0.3524 | Yes |
| 19 | SRC | na | 4033 | 0.235 | 0.3398 | Yes |
| 20 | MYH4 | na | 4076 | 0.234 | 0.3509 | Yes |
| 21 | ACTN1 | na | 4698 | 0.211 | 0.3441 | Yes |
| 22 | CLDN6 | na | 5033 | 0.201 | 0.3450 | Yes |
| 23 | CLDN18 | na | 5084 | 0.198 | 0.3540 | Yes |
| 24 | MYL9 | na | 5666 | 0.180 | 0.3468 | Yes |
| 25 | ACTN3 | na | 5743 | 0.178 | 0.3539 | Yes |
| 26 | MPDZ | na | 5764 | 0.178 | 0.3627 | Yes |
| 27 | CLDN17 | na | 5912 | 0.173 | 0.3676 | Yes |
| 28 | CLDN10 | na | 6231 | 0.164 | 0.3671 | Yes |
| 29 | MYH7B | na | 6460 | 0.158 | 0.3688 | Yes |
| 30 | TJAP1 | na | 7216 | 0.138 | 0.3542 | No |
| 31 | MYH6 | na | 7367 | 0.134 | 0.3569 | No |
| 32 | NRAS | na | 7693 | 0.126 | 0.3541 | No |
| 33 | EPB41L3 | na | 7881 | 0.121 | 0.3551 | No |
| 34 | PRKCA | na | 7938 | 0.120 | 0.3598 | No |
| 35 | CTNNA3 | na | 8333 | 0.112 | 0.3543 | No |
| 36 | RRAS | na | 8436 | 0.109 | 0.3571 | No |
| 37 | PPP2CB | na | 8608 | 0.106 | 0.3578 | No |
| 38 | GNAI1 | na | 9135 | 0.096 | 0.3477 | No |
| 39 | PPP2CA | na | 9540 | 0.090 | 0.3407 | No |
| 40 | PARD6A | na | 9543 | 0.090 | 0.3454 | No |
| 41 | RRAS2 | na | 9773 | 0.085 | 0.3432 | No |
| 42 | CDK4 | na | 9992 | 0.082 | 0.3412 | No |
| 43 | CLDN15 | na | 10097 | 0.080 | 0.3424 | No |
| 44 | MYL2 | na | 10210 | 0.078 | 0.3433 | No |
| 45 | PPP2R2A | na | 10512 | 0.073 | 0.3384 | No |
| 46 | MYH10 | na | 11002 | 0.066 | 0.3278 | No |
| 47 | MYH7 | na | 11734 | 0.056 | 0.3096 | No |
| 48 | TJP3 | na | 11828 | 0.055 | 0.3098 | No |
| 49 | CASK | na | 12232 | 0.050 | 0.3008 | No |
| 50 | PPP2R1A | na | 12268 | 0.049 | 0.3024 | No |
| 51 | CSNK2B | na | 13092 | 0.039 | 0.2807 | No |
| 52 | CSNK2A2 | na | 13289 | 0.037 | 0.2770 | No |
| 53 | CLDN1 | na | 13367 | 0.037 | 0.2767 | No |
| 54 | MYH9 | na | 13965 | 0.031 | 0.2610 | No |
| 55 | YES1 | na | 14068 | 0.030 | 0.2597 | No |
| 56 | PRKCH | na | 14323 | 0.027 | 0.2538 | No |
| 57 | VAPA | na | 14430 | 0.026 | 0.2521 | No |
| 58 | MYH2 | na | 14556 | 0.025 | 0.2498 | No |
| 59 | HRAS | na | 14559 | 0.025 | 0.2511 | No |
| 60 | RAB13 | na | 14675 | 0.024 | 0.2490 | No |
| 61 | MAGI3 | na | 15271 | 0.019 | 0.2328 | No |
| 62 | RHOA | na | 15382 | 0.018 | 0.2305 | No |
| 63 | AKT1 | na | 15787 | 0.014 | 0.2196 | No |
| 64 | CLDN16 | na | 15978 | 0.013 | 0.2148 | No |
| 65 | MYL12A | na | 16077 | 0.012 | 0.2126 | No |
| 66 | MYH11 | na | 16745 | 0.007 | 0.1937 | No |
| 67 | ACTG1 | na | 16994 | 0.005 | 0.1868 | No |
| 68 | MYH13 | na | 17626 | -0.001 | 0.1686 | No |
| 69 | LLGL1 | na | 17781 | -0.002 | 0.1643 | No |
| 70 | F11R | na | 17825 | -0.003 | 0.1632 | No |
| 71 | CDC42 | na | 17874 | -0.003 | 0.1619 | No |
| 72 | PRKCD | na | 17924 | -0.003 | 0.1607 | No |
| 73 | MYL12B | na | 18193 | -0.006 | 0.1532 | No |
| 74 | CTNNB1 | na | 18214 | -0.006 | 0.1530 | No |
| 75 | CRB3 | na | 18444 | -0.008 | 0.1468 | No |
| 76 | CTNNA1 | na | 18820 | -0.011 | 0.1365 | No |
| 77 | CLDN9 | na | 18876 | -0.012 | 0.1356 | No |
| 78 | PRKCI | na | 19141 | -0.014 | 0.1287 | No |
| 79 | GNAI2 | na | 19897 | -0.021 | 0.1080 | No |
| 80 | PARD3 | na | 20314 | -0.025 | 0.0972 | No |
| 81 | PARD6G | na | 20864 | -0.030 | 0.0830 | No |
| 82 | YBX3 | na | 21047 | -0.032 | 0.0794 | No |
| 83 | PRKCZ | na | 21192 | -0.034 | 0.0770 | No |
| 84 | CLDN4 | na | 21632 | -0.039 | 0.0664 | No |
| 85 | ACTN4 | na | 21725 | -0.040 | 0.0658 | No |
| 86 | PPP2R2D | na | 22091 | -0.044 | 0.0576 | No |
| 87 | TJP2 | na | 22117 | -0.044 | 0.0592 | No |
| 88 | CLDN19 | na | 22264 | -0.046 | 0.0574 | No |
| 89 | CLDN8 | na | 22405 | -0.047 | 0.0558 | No |
| 90 | ACTB | na | 22412 | -0.047 | 0.0581 | No |
| 91 | MYH14 | na | 22684 | -0.051 | 0.0530 | No |
| 92 | CTNNA2 | na | 22741 | -0.051 | 0.0541 | No |
| 93 | CGN | na | 22760 | -0.052 | 0.0562 | No |
| 94 | SPTAN1 | na | 23105 | -0.056 | 0.0493 | No |
| 95 | EXOC4 | na | 23130 | -0.056 | 0.0515 | No |
| 96 | CTTN | na | 23182 | -0.057 | 0.0531 | No |
| 97 | CLDN5 | na | 23339 | -0.059 | 0.0517 | No |
| 98 | PTEN | na | 23399 | -0.060 | 0.0532 | No |
| 99 | PRKCQ | na | 23761 | -0.066 | 0.0462 | No |
| 100 | LLGL2 | na | 23832 | -0.067 | 0.0478 | No |
| 101 | CLDN11 | na | 23906 | -0.068 | 0.0492 | No |
| 102 | CSNK2A1 | na | 24568 | -0.078 | 0.0343 | No |
| 103 | TJP1 | na | 24758 | -0.081 | 0.0331 | No |
| 104 | PPP2R2C | na | 24813 | -0.082 | 0.0359 | No |
| 105 | KRAS | na | 25083 | -0.087 | 0.0326 | No |
| 106 | MYH3 | na | 25442 | -0.093 | 0.0272 | No |
| 107 | EXOC3 | na | 25790 | -0.100 | 0.0224 | No |
| 108 | AMOTL1 | na | 26002 | -0.104 | 0.0218 | No |
| 109 | EPB41L1 | na | 26329 | -0.111 | 0.0183 | No |
| 110 | PPP2R2B | na | 26443 | -0.114 | 0.0210 | No |
| 111 | CLDN7 | na | 26468 | -0.114 | 0.0263 | No |
| 112 | ASH1L | na | 26786 | -0.121 | 0.0235 | No |
| 113 | CLDN3 | na | 27052 | -0.127 | 0.0225 | No |
| 114 | AKT2 | na | 27111 | -0.128 | 0.0276 | No |
| 115 | MYL5 | na | 27140 | -0.129 | 0.0336 | No |
| 116 | SYMPK | na | 27732 | -0.144 | 0.0241 | No |
| 117 | OCLN | na | 27893 | -0.148 | 0.0272 | No |
| 118 | GNAI3 | na | 28235 | -0.158 | 0.0257 | No |
| 119 | EPB41 | na | 28510 | -0.164 | 0.0264 | No |
| 120 | PPP2R1B | na | 28524 | -0.165 | 0.0347 | No |
| 121 | PRKCG | na | 28978 | -0.178 | 0.0310 | No |
| 122 | MYH15 | na | 30036 | -0.212 | 0.0116 | No |
| 123 | RAB3B | na | 30135 | -0.215 | 0.0202 | No |
| 124 | CLDN23 | na | 30304 | -0.222 | 0.0270 | No |
| 125 | MYH1 | na | 31917 | -0.289 | -0.0044 | No |
| 126 | CLDN20 | na | 33117 | -0.377 | -0.0192 | No |
| 127 | CLDN22 | na | 34724 | -1.248 | 0.0001 | No |
Table: GSEA details [plain text format]

  

Fig 2: KEGG\_TIGHT\_JUNCTION      
 Blue-Pink O' Gram in the Space of the Analyzed GeneSet

  

Fig 3: KEGG\_TIGHT\_JUNCTION: Random ES distribution      
 Gene set null distribution of ES for **KEGG\_TIGHT\_JUNCTION**
